# Functionalization of Sodium Caseinate for Production of Neat Films: Effects of Casein Crosslinking Induced by Heating at Alkaline pH or Light Exposure

**DOI:** 10.3390/foods14162764

**Published:** 2025-08-08

**Authors:** Paolo D’Incecco, Stefano Gerna, Marta Sindaco, Luisa Pellegrino, Alberto Barbiroli, Veronica Rosi, Sara Limbo

**Affiliations:** Department of Food, Environmental and Nutritional Sciences (DeFENS), Università degli Studi di Milano, Via G. Celoria 2, 20133 Milan, Italy; stefano.gerna@unimi.it (S.G.); marta.sindaco@unimi.it (M.S.); luisa.pellegrino@unimi.it (L.P.); alberto.barbiroli@unimi.it (A.B.); veronica.rosi@unimi.it (V.R.); sara.limbo@unimi.it (S.L.)

**Keywords:** sodium caseinate, crosslinking, di-tyrosine, lysinoalanine, caseinate film, thermal treatment, FTIR, mechanical properties

## Abstract

This study explored the functionalization of sodium caseinate (NaCas) using environmentally friendly approaches to improve the mechanical and structural properties of the derived films. NaCas functionalization was achieved through casein crosslinking using two approaches: (i) thermal treatment at an alkaline pH to induce the formation of lysinoalanine (LAL) and (ii) riboflavin-mediated photo-oxidation to induce the formation of di-tyrosine (di-Tyr). Starting from NaCas (not functionalized, control) obtained from pasteurized milk, three functionalized NaCas samples were prepared: one sample crosslinked by LAL, and two samples crosslinked by di-Tyr formed under LED light either with or without riboflavin. The amount of crosslinking was evaluated in the acid hydrolysates through HPLC methods using either fluorescence (di-Tyr) or MS (LAL) detection. Heat treatment at pH 9 induced the formation of up to 3540 µg of LAL/g casein, whereas LED light exposure in the presence of riboflavin promoted the formation of up to 500 µg of di-Tyr/g casein. The formation of crosslinks at the intermolecular level, which resulted in protein aggregation, was detected by SDS-PAGE. Films were obtained by mixing the water solutions of the four NaCas samples with glycerol as the plasticizer and casting them. The FTIR spectra revealed that the formation of crosslinks also induced changes in the secondary structure of NaCas, which were conserved in the derived films. Mechanical testing demonstrated that di-Tyr crosslinks enhanced film ductility, while LAL crosslinks increased tensile strength and stiffness.

## 1. Introduction

Sodium caseinate (NaCas), a derivative of milk, is produced by dissolving acid-precipitated casein with sodium hydroxide, followed by drying. Casein is a family of different proteins that are present in milk as complex aggregates or micelles stabilized by clusters of calcium phosphate. Caseins have a random coil structure and can participate in many molecular interactions through hydrogen bonding, electrostatic interactions, and hydrophobic interactions [1]. Like other natural biopolymers, casein has attracted research interest as a potential alternative to synthetic polymers for producing films and coatings [2,3]. Due to their high proportion of polar groups, films obtained from NaCas have good barrier properties against non-polar substances such as oxygen, carbon dioxide, and volatile organic compounds. On the other hand, due to their relatively high hydrophilicity, such films are not good barriers to water vapor. To address this drawback, NaCas solutions are often coated onto a support such as paperboard or blended with reinforcing components [4]. However, this approach does not align with the recent trend of adopting mono-material film packaging that can be more effectively recycled [5]. Casein-based films are rather brittle and tend to desiccate easily. Their mechanical properties, such as tensile strength and flexibility, are commonly improved by adding a plasticizer into the formulation [6]. In our recent study, we prepared experimental films from solutions of NaCas obtained from partly skimmed pasteurized milk. The solutions were mixed with glycerol as a plasticizer and subjected to ultrasound treatment before casting [7]. Depending on the laboratory production conditions, the obtained films had a residual milk fat content ranging from 1 to 25% (*w*/*w*). The presence of milk fat improved the properties of these films compared with the film prepared using a fat-free commercial NaCas. However, there is still potential for further improvement.

A strategy for improving the mechanical and barrier properties of NaCas films is crosslinking the casein molecules using different approaches before producing the films [8,9,10]. Creating crosslinks between the protein chains may result in a strengthened three-dimensional network, leading to modified techno-functional properties of the derived films, including increased water resistance. This approach is more cost-effective compared to novel approaches such as those using nanoparticles as fillers [11]. The crosslinking process involves the formation of chemical bonds between two amino acid residues of a single (intramolecular) or different (intermolecular) protein chains. Most of the focus has been on covalent crosslinks since they are normally irreversible and, when formed intermolecularly, induce the formation of polymers and stable aggregates [12,13,14,15]. The stability of the modifications induced in a biomaterial is important for many industrial applications. The susceptibility of a protein to crosslinking primarily depends on its structure; for example, the two reacting amino acid residues in the protein backbone must be able to come in close proximity to form the crosslink.

Among the numerous approaches for crosslinking caseins, those based on the Maillard reaction have been extensively investigated. While this reaction mainly entails protein glycation by simple sugars, the direct formation of crosslinked species involves two free amino groups of casein that are bridged by a di-aldehyde, such as glutaraldehyde, malondialdehyde, glyoxal, or formaldehyde, with all these compounds inducing browning but having recognized toxicities [10]. Other studies investigated other agents for inducing casein crosslinking, including tannic acid [16], genipin [9], and transglutaminase [17].

In the present study, we evaluated two different approaches for achieving casein crosslinking without the addition of a reagent. Both approaches are based on well-established chemical pathways that were previously investigated in proteins in different fields. At the same time, the two types of crosslinks were intentionally kept at low levels in order to improve the functional properties of the derived films without compromising their possible use as food contact films and their biodegradability.

The first approach for casein crosslinking involves a photo-oxidation reaction that may follow different pathways depending on the oxygen availability [18,19], which can be catalyzed by riboflavin. In milk, endogenous riboflavin (RF) acts as a photosensitizer: it is excited by light to the triplet state (^3^RF *), which readily transfers electrons to induce oxidation of a substrate, primarily tyrosine residues in proteins [20]. Radicals of tyrosine in the casein backbone become crosslinked by forming di-tyrosine (di-Tyr) bonds (pathway 1). Under low oxygen conditions, this pathway competes with the self-reaction of tyrosine, which is mediated by singlet molecular oxygen, leading to the formation of crosslinks (pathway 2). The formation of di-Tyr has been observed in individual casein fractions [19], in infant formula [21], and in pasteurized milk stored in a commercial display counter [22].

The second approach for casein crosslinking involves the β-elimination reaction of selected amino acid residues, leading to the formation of lysinoalanine (LAL) crosslinks. LAL is an unnatural amino acid that forms in proteins upon heat treatment under alkaline conditions. Its formation occurs through a two-step mechanism: a β-elimination reaction at a cysteine or phosphoserine residue forms a dehydroalanine residue that then reacts with the ε-amino group of a lysine residue [15]. The second step of the reaction is strongly suppressed in the presence of reducing sugars because the Maillard reaction competes for the same amino acid [23]. The highest levels of LAL have been found in low-lactose dairy products such as casein and caseinate powders [14,24,25].

In the present study, these two approaches were applied to produce functionalized NaCas that were further processed into films through casting. To evaluate the effects of the two functionalization treatments, the films were prepared from NaCas obtained from pasteurized milk, and no other components were added except for glycerol (used as the plasticizing agent). Taking advantage of the stability of both di-Tyr and LAL against acid hydrolysis, these compounds were quantified in the hydrolysate using chromatographic methods. The formation of multimeric species of caseins in the functionalized NaCas powders was assessed using SDS-PAGE, while the properties of the derived films were tested using FTIR spectroscopy and by evaluating their tensile strength and elongation at break. This study aimed to clarify whether low amounts of the selected crosslinks can improve the mechanical properties of films fabricated using casein recovered from pasteurized milk as the sole structural component.

## 2. Materials and Methods

### 2.1. Preparation of Sodium Caseinate Powder

**Figure 1:**
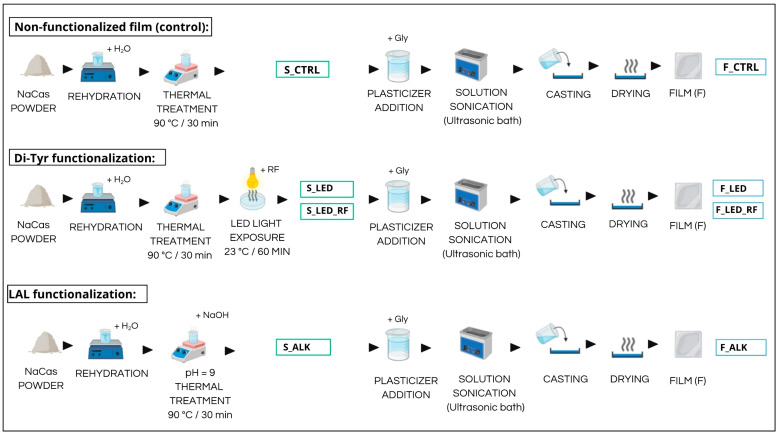
Experimental design for NaCas functionalization and film production. **Top**: Production of films from non-functionalized NaCas (control); **middle**: production of films from NaCas functionalized by exposing the solution to LED light; **bottom**: production of films from NaCas functionalized by heating at alkaline pH. The sampling points and the corresponding sample codes for the NaCas solutions (S) and films (F) are indicated.

A large batch (6 L) of partly skimmed (fat 1.5 g/100 mL) pasteurized milk was obtained from a local shop. Pasteurized milk was used to avoid the presence of denatured whey proteins bound to casein. The fat content of the milk was further reduced through centrifugation at 8000× *g* for 20 min at 40 °C, following the method in [7]. The oily supernatant was removed with a pipette, and the skimmed milk was poured into a beaker. The pH of the milk was adjusted to 4.6 through the dropwise addition of 1 N HCl, and the whey was separated through centrifugation at 4000× *g* for 15 min at 4 °C. The pelleted casein was recovered and suspended in MilliQ water (Waters, Milan, Italy) by stirring with a magnetic stirrer for 15 min. The dispersion was centrifuged again, and the washing step was repeated twice. The pellet was dispersed in a small amount of MilliQ water (approx. 100 g wet pellet/150 mL water), and the dispersion was adjusted to pH 7.0 through the dropwise addition of 1 N NaOH under gentle stirring until complete solubilization was achieved. The solution was filtered using paper filter, transferred into a stainless-steel tray, and freeze-dried (Lio10P, 5Pascal, Milan, Italy). The freeze-dried NaCas was finely ground and stored in a sealed jar until use. The samples used in this study were all prepared from this batch of powder following the experimental design outlined in Figure 1. The composition of the powder is reported in Table S1.

### 2.2. Preparation of Control NaCas Solution (Untreated)

The NaCas solution was prepared as described by Gerna et al. [7] and was used as the control (S_CTRL). Briefly, the NaCas powder was dissolved in MilliQ water (10% protein, *w*/*v*) by stirring for 10 min. Afterward, the solution was heated to 90 °C, stirred at this temperature for 30 min using a magneto-thermic stirrer (C-MAG HS 4, IKA, Staufen, Germany), and finally stirred for an additional 15 min at room temperature to allow it to cool down. An aliquot of the S_CTRL solution was freeze-dried for the analyses.

### 2.3. Preparation of LAL-Functionalized NaCas Solution

The NaCas powder was dissolved in MilliQ water (10% protein, *w*/*v*) by stirring for 10 min. Afterward, the solution was carefully adjusted to pH 9 through the dropwise addition of 1 N NaOH, incubated at 90 °C for 30 min under stirring, and finally stirred for an additional 15 min at room temperature to allow it to cool down (S_ALK). An aliquot of the S_ALK solution was freeze-dried for the analyses.

### 2.4. Preparation of Di-Tyr-Functionalized NaCas Solutions

A 10% NaCas solution (*w*/*v*, in water) was prepared and heated as described for the control. Then, it was divided into two aliquots, which were both subjected to LED illumination: one aliquot was illuminated as is (S_LED), and to the other, riboflavin (RF) was added as a photosensitizer (S_LED_RF) before illumination. The RF solution (pure riboflavin from Sigma-Aldrich, St. Louis, MO, USA) was prepared in MilliQ water by stirring and 30 s of sonication to achieve complete solubilization, which was then added to the NaCas solution to reach a final concentration of 35 µmol/L [19]. The two solutions (30 mL) were poured into separate Petri dishes (area of 113 cm^2^) that were positioned under an LED lamp (Multispectrum 900000920, Atena Lux, Venezia, Italy) at a distance of 38 cm, as shown in Figure S1A. During the 60 min illumination, the solutions were gently mixed every 15 min to ensure homogeneous exposure. The spectral irradiance of the lamp is shown in Figure S1B. After light exposure, aliquots of the S_LED and S_LED_RF solutions were freeze-dried for the analyses.

### 2.5. Determination of the Crosslinks

#### 2.5.1. Acid Hydrolysis

The freeze-dried NaCas samples from both the control and the functionalized solutions were subjected to acid hydrolysis, and the amounts of the two crosslinks (di-Tyr and LAL) in the hydrolysates were determined. An aliquot of powder corresponding to 10 mg protein was weighed in a glass vial, 8 mL of 6 N HCl was added, and the solution was hydrolyzed under vacuum at 110 °C for 23 h. The hydrolysate was filtered using a paper filter (Whatman, Chicago, IL, USA) and subjected to crosslink analyses.

#### 2.5.2. Determination of LAL Content by LC-MS

To determine the LAL content, 2 mL of the hydrolysates were evaporated at 40 °C and resuspended in 1 mL of MilliQ water, centrifuged at 10,000× *g* for 5 min, and then diluted 1:25 with ultra-pure water (analytical grade for LC-MS). The LC-MS analysis method of Akıllıoğlu et al. [26] was used with modifications with an Agilent 1290 Infinity II LC connected to an Agilent 6495 LC/TQ (Agilent, Santa Clara, CA, USA). Chromatographic separation was performed on a Kinetex Hilic column (Phenomenex, Torrance, CA, USA) (100 mm × 2.1 mm i.d., 1.7 µm particle size) at a flow rate of 0.4 mL/min with a binary gradient of solvent A (0.1% formic acid and 1 M ammonium formiate in water) and solvent B (90% acetonitrile with 0.1% formic acid and 1 M ammonium formiate). The elution gradient was as follows: 20% solvent B for 2 min, increase to 25% over 1.5 min, increase to 95% over 1 min, and held at 95% for 1.5 min. The column was then equilibrated with 20% B for 5 min before the subsequent injection. The injection volume was 5 µL. A calibration curve was obtained by analyzing LAL solutions with four different concentrations (0.50, 0.75, 1.0, and 1.5 µg/mL) prepared from pure LAL (lysinoalanine 2HCl, F-1195.0050, Bachem, Bubendorf, Switzerland). The data are expressed as µg/g protein and μmol/g protein.

Mass spectrometry spectra were collected in the positive ion and MRM (Multiple Reaction Monitoring) modes under the following conditions: precursor ion (H^+^)—234.2 *m*/*z*; fragment ion—84.1 *m*/*z*; normalized collision energy (NCE)—28.

#### 2.5.3. Determination of Di-Tyr Content by HPLC

The di-Tyr content was measured using the method developed by D’Incecco et al. (2024) [22]. The hydrolysate (2 mL) was evaporated at 40 °C using a Multivapor P-12 (Büchi Italia, Cornaredo, MI, Italy), suspended in 250 µL of 0.1% (*v*/*v*) trifluoroacetic acid (TFA) in MilliQ water, and analyzed by HPLC. The equipment was an Alliance 2695 HPLC system (Waters, Milan, Italy) coupled with a Hitachi L2480 fluorescence detector (VWR, Milan, Italy) set at 285 nm for excitation and 400 nm for emission. Chromatographic separation was performed on a Platinum EPS C18 column (Grace Alltech, Fisher Scientific, Segrate, Italy) (4.6 mm i.d. × 150 mm, 100 Å pore size, 3 µm particle size), which was maintained at 30 °C, and eluted at a flow rate of 0.8 mL/min with a binary gradient of solvent A (0.1% (*v*/*v*) TFA in MilliQ water) and solvent B (0.1% (*v*/*v*) TFA in acetonitrile). The elution gradient was as follows: 2% solvent B for 1 min, increase to 5% over 14 min, increase to 98% over 10 min, and held at 98% for 5 min (washing step). The column was then equilibrated with 2% B for 5 min before the subsequent injection. The injection volume was 10 µL. Empower 2 software (Waters) was used to process the chromatographic data. The di-Tyr content was calculated using a 4-point calibration curve prepared from pure di-Tyr (Toronto Research Chemicals, Vaughan, ON, Canada) and was expressed as µg/g protein and μmol/g protein.

### 2.6. Sodium Dodecyl Sulfate–Polyacrylamide Gel Electrophoresis (SDS-PAGE)

The formation of covalent aggregates in both the control and functionalized NaCas samples was evaluated by SDS-PAGE under reducing conditions. The freeze-dried NaCas samples (1 mg) were suspended in 100 μL of water and 100 μL of denaturing buffer (0.125 M Tris-HCl (pH 6.8), 50% (*v*/*v*) glycerol, 1.7% (*w*/*v*) SDS, 0.01% (*w*/*v*) bromophenol blue, and 1% (*v*/*v*) 2-mercaptoethanol). Subsequently, the suspension was incubated at 100 °C for 10 min, and then 4 µL of the denatured solution was loaded into a 12.5% polyacrylamide gel. The electrophoretic run was performed at pH 8.8 (0.025 M Tris-HCl, 0.192 M glycine, and 0.1% (*w*/*v*) SDS) in a Miniprotean II electrophoresis cell (Bio-Rad Laboratories, Hercules, CA, USA), and then the gels were stained with Coomassie Blue. Low-Range SDS-PAGE Standards, 14–97 kDa (Bio-Rad), were used as protein markers, and acid casein from raw milk was used as a reference sample. Densitometric analysis of the gel was carried out using the ImageMasterTM 1D Elite v 4.00 software (Amersham Pharmacia Biotech, Cologno Monzese, Italy).

### 2.7. Preparation and Characterization of NaCas Films

The film-forming NaCas solutions were prepared as described by Gerna et al. [7]. Briefly, bi-distilled glycerol (analytical grade, VWR Chemicals, Milan, Italy) was added to the S_CTRL, S_ALK, S_LED, and S_LED_RF solutions at a concentration of 33.3% (*w*/*w*) under stirring for 10 min to ensure homogeneous dispersion. The resulting solutions (30 mL) underwent 10 min of continuous sonication in an ultrasonic bath (Transonic T310, GEASS, Turin, Italy), operating at a frequency of 37 kHz and a constant power of 34.5 W, to remove any air bubbles. The solutions were cast into Teflon molds measuring 10 × 10 cm at a ratio of 0.1 mL/cm^2^, as described by Gerna et al. [7]. The molds were left to dry at room temperature (21–23 °C) for 48 h. After the drying period, the films (F_CTRL, F_ALK, F_LED, and F_LEF_RF) were peeled off of the molds, individually sandwiched between two sheets of aluminum foil, stored in a polystyrene box at 4 °C, and analyzed within 3 days. Three different films were prepared from each solution.

#### 2.7.1. Film Thickness

Film thickness was measured using a digital Mitutoyo QuantuMike Digimatic micrometer (Takatsu-ku, Kawasaki, Kanagawa, Japan). For each film, the average of nine values, measured at random positions, was recorded as the film thickness.

#### 2.7.2. Fourier-Transform Infrared (FTIR) Spectroscopy

FTIR spectroscopy analysis of the films was performed using a Nicolet i550 FT-IR instrument (Thermo Fisher Scientific, Madison, WI, USA) equipped with a germanium attenuated total reflection (ATR) module. Spectra were recorded from 4000 cm^−1^ to 650 cm^−1^ at ambient temperature, at a resolution of 4 cm^−1^, and as an accumulation of 40 scans. Three replicates for each sample were analyzed. After the baseline corrections, the spectra were smoothed using the 25-point Savitsky–Golay method, and Fourier deconvolution (FD) was performed using Omnic software (v.9, Thermo Fisher Scientific, Madison, WI, USA). In the Amide I region, several regions were analyzed including 1699–1682 cm^−1^ (intermolecular/aggregated β-sheets); 1681–1662 cm^−1^ (β-turns); 1662–1645 cm^−1^ (α-helices); 1645–1638 cm^−1^ (random coils); 1637–1615 cm^−1^ (intramolecular β-sheets); and 1614–1608 cm^−1^ (side chains) [27]. Each peak in the FD spectra with a high percentage was integrated.

#### 2.7.3. Mechanical Properties

The mechanical properties of the films, namely tensile strength (TS) and elongation at break (EAB), were evaluated following the ASTM D882-18 protocol [28] and the conditions described by Gerna et al. [7]. The tests were carried out using an HDplusC Texture Analyser (Stable Micro Systems, Godalming, UK) equipped with suitable grips for TS measurements. The films were preliminarily conditioned at 50% RH and 20 °C, and the tests were carried out at room temperature. Rectangular strips of film (20 × 100 mm) were clamped with an initial grip separation of 60 mm. A crosshead speed of 20 mm min^−1^ was applied until the film broke. For each film, the TS (MPa) and EAB (%) were calculated from the force–strain curves as the average of at least eight tests.

### 2.8. Statistical Analysis

One-way ANOVA was carried out using the SPSS Win 12.0 software version 27 (IBM Corp., Chicago, IL, USA), and the samples were considered statistically different if *p* < 0.05.

## 3. Results and Discussion

### 3.1. Lysinoalanine (LAL) Formation in NaCas Solutions Heated Under Alkaline Conditions

LAL can form from the nucleophilic addition of the ε-amino group of a lysine to a dehydroalanine residue [15]. In casein, which has a low cysteine content, dehydroalanine can only be generated from phosphoseryl residues through a β-elimination reaction. To accurately measure LAL levels, a method based on LC-MS was adopted. The levels of LAL detected in the experimental samples are shown in Table 1. In order to evaluate the cross-linking capacity, the molar ratio between the LAL and casein was calculated. Considering the average molecular weight of the casein subunits, 1 g of casein corresponds to about 43 µmol.

All of the samples contained LAL. This was not surprising because, upon heating, LAL formation occurs even at neutral pH [29]. To achieve proper solubilization of acid casein when preparing the NaCas samples, a rather strong heat treatment (90 °C for 30 min) at pH 7 was necessary. The control sample (S_CTRL) and the NaCas exposed to LED light had comparable levels of LAL (867 and 907 µg/g protein, respectively), with an LAL/casein molar ratio of about 0.1:1. Interestingly, the level of LAL was slightly lower in the powder exposed to LED light in presence of RF.

When the heat treatment of the solution was carried out at pH 9 (S_ALK), the obtained amount of LAL was 3540 µg/g protein, which is 4 times higher than that obtained for the sample heated at pH 7 (S_CTRL) (15.86 µmol/g protein; 0.37:1 molar ratio compared to casein). The presence of LAL in commercial NaCas has been previously reported, with levels of up to 350 µg/g protein [24], 500 µg/g [30], and 770 µg/g [25]. The amounts of LAL were much higher here, possibly due to the more specific analytical method we utilized (LC-MS). Moreover, a thorough washing of the casein was carried out to remove lactose. This was performed to avoid glycation of lysine residues, which were thus fully available for LAL formation during the subsequent processing. Previous studies reported that LAL formation in casein is promoted by heating, alkaline pHs, and high alkali concentrations, while a marked decrease occurs under extreme heat conditions (e.g., 95 °C for 1 h) [29]. At lower temperatures, the rates of LAL formation were lower, but the final levels were much higher. However, no further attempts to obtain very high levels of LAL in the NaCas were made since we were focused on preserving the biodegradability of the derived films while enhancing their functional properties.

Another aspect that could be worth investigating in the formation of LAL is the effect of the sonication treatment that was adopted to reduce the formation of air bubbles during the subsequent casting and drying of the film [7]. Interestingly, it has been reported that sonication can prevent the formation of LAL in alkali-treated proteins since the cavitation forces generated by ultrasound waves modify their secondary structure. This effect is mainly observed in proteins rich in sulfhydryl groups, such as rice dregs [31] or rapeseed [32], but could also occur in casein. This treatment could either contribute to the accessibility of reactive lysine residues, ultimately increasing the formation of intramolecular LAL crosslinks, or improve molecule dispersion, thus promoting the formation of intermolecular LAL crosslinks. This latter effect could be helpful for improving the mechanical stability of NaCas-based films without requiring the addition of an alkali.

### 3.2. Di-Tyr Formation in NaCas Solutions Exposed to LED Light

Di-Tyr determination was carried out using the HPLC method with fluorescence detection developed by D’Incecco et al. (2024) [22]. Under the adopted chromatographic conditions, di-Tyr elutes as a main peak with a small shoulder, likely due to a minor stereoisomer (Figure S2). No di-Tyr was detected in the control solution (S_CTRL) (not functionalized), nor in the solution that underwent heat treatment at an alkaline pH (S_ALK) (Table 2). Direct exposure to LED light for 60 min induced the formation of 4 µg of di-Tyr per g protein in S_LED, while the amount increased up to 501 µg/g (1.39 µmol/g protein, molar ratio with casein 0.03:1) when RF was added (S_LED_RF). This aligns with the well-established role of RF as a photo-activator of oxidative modifications to casein molecules, leading to the formation of crosslinks, such as di-Tyr, among others. The procedure adopted here to recover casein from milk likely led to the depletion of endogenous RF in the derived NaCas powder since thorough washes were carried out. This explains the lack of di-Tyr in the S_CTRL and S_ALK samples and the trace level in S_LED despite the exposure to LED light.

Riboflavin is naturally present in milk at a concentration of 1.8–2 mg/L (4.8–5.3 µmol/L) [33,34]. In a recent study, we demonstrated that these natural levels of RF can induce the formation of up to 250–300 µg of di-Tyr/g protein in pasteurized milk in clear PET bottles exposed to fluorescent light in a display cabinet for 7 days [22]. To induce extensive crosslinking in NaCas, the concentration of RF in the NaCas solution was increased up to 35 µmol/L (about 7-fold higher levels than those in natural milk). In our samples, this addition promoted a more than 100-fold increase in the di-Tyr level compared to the solution without RF. Based on the study of Fuentes-Lemus et al. [19], in solutions of pure α-casein and β-casein with 35 µmol/L RF that were exposed to light for 60 min, di-Tyr was the most abundant cross-link. Dalsgaard et al. [18] showed that the addition of 13 µmol/L RF to β-casein, β-lactoglobulin, or BSA solutions induced a sharp increase in di-Tyr levels during the first 10 min of illumination, and the amount was about three times higher in casein than in the globular whey proteins, likely due to the flexible structure of the former. Unfortunately, the di-Tyr data in the literature are difficult to compare because of the different units and analytical methods used. Chen et al. [21] reported di-Tyr values in the range of 1–2 mg/g protein in commercial samples of infant formula containing both casein and whey proteins. It was not mentioned whether these samples had added vitamins and thus might have a high RF content, although heat-sensitive ingredients are usually dry-blended at the end of the manufacturing process [35].

Overall, these data indicate that the addition of a photo-activator, specifically RF, strongly increases the amount of di-Tyr, which could modify the functional properties of NaCas films. It should be underlined that RF is fully compatible with an edible film, and the obtained amount of di-Tyr is only two times that found in commercial pasteurized milk after 7 days of storage on the market.

Nevertheless, the amount of di-Tyr in S_LED_RF (501 µg/g protein) corresponds to 2.8 µmol of crosslinked tyrosine out of the 340 µmol of tyrosine per g of casein that is potentially available, suggesting a limited reactivity of the system. Fuentes-Lemus et al. [19] demonstrated that the formation of di-Tyr residues through photo-oxidation is higher under anaerobic conditions since oxygen can react with the intermediates, leading to different final products. Therefore, the LED illumination of a sample spread in a thin layer in a Petri dish could inhibit the reaction, maximizing the gas exchange surface with the surrounding environment. Furthermore, as discussed above, all the experimental samples already contained some LAL that could have partly prevented the close proximity of two tyrosine residues that is necessary for di-Tyr formation. Since our focus was on intermolecular di-Tyr crosslinks, other approaches were implemented to investigate this aspect.

### 3.3. SDS-PAGE of Functionalized NaCas Solutions

The formation of aggregates in the functionalized NaCas was evaluated by SDS-PAGE under reducing conditions. Thanks to its ability to preserve the covalent bonds formed through LAL and di-Tyr, electrophoresis is a suitable separation technique to evaluate the susceptibility of casein to crosslinking reactions [13].

**Figure 2:**
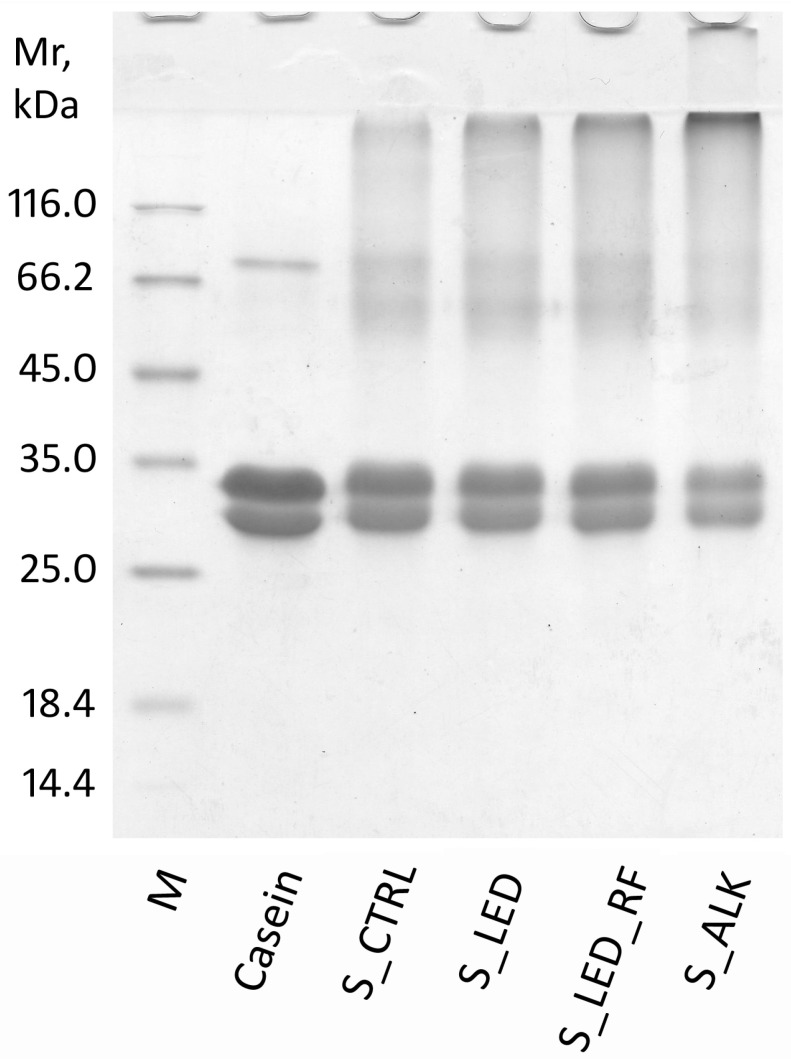
SDS-PAGE of casein samples under reducing conditions. M: MW markers; Casein: casein from raw milk; S_CTRL: control NaCas solution (non-functionalized); S_LED: NaCas solution exposed to LED light; S_LED_RF: NaCas solution exposed to LED light in the presence of riboflavin; S_ALK: NaCas solution heat-treated at alkaline pH.

The electrophoretic separation is shown in Figure 2. The samples were loaded with equal amounts of protein to allow for a direct comparison of the intensities of the protein bands. The 12.5% acrylamide gel used allowed for the separation of casein into two main bands, with the upper one being attributable to α_s_-caseins, while the lower one corresponds to β- and k-caseins [36]. As expected, acid casein separated from milk did not contain aggregates, and the subunits showed an apparent molecular weight of about 30–33 kDa, as expected for monomers. The band at about 80 kDa is likely due to residual lactoferrin since the acid casein was not washed after the precipitation.

In contrast, the non-functionalized NaCas sample (S_CTRL) showed the presence of aggregates. The bands attributable to the monomeric forms decrease in intensity (S_CTRL band volume was 75% of that of native casein) (Table S2), and new bands appeared around the molecular weight of 66 kDa, which could be dimeric forms of the casein subunit. Furthermore, an accumulation of protein was localized in the upper part of the gel, on the border between the stacking and the separating gel, which was attributable to polymeric forms of such a large size that they are unable to migrate into the gel. The presence of these aggregates is consistent with the presence of intermolecular crosslinks due to the formation of LAL during the thermal treatment to form NaCas, as previously highlighted. The 0.1:1 molar ratio of LAL to casein is consistent with this result.

The role of LAL in the formation of intermolecular crosslinks is highlighted by the electrophoretic trace of the S_ALK sample (Figure 2, line 5), which showed a notable reduction in the monomeric forms and an accumulation of high-molecular-weight aggregates (S_ALK band volume was 46% of that of native casein) (Table S2). It is noteworthy that there was little accumulation of dimers compared to the higher-molecular-weight polymeric forms.

Under the experimental conditions adopted in this study, no formation of di-Tyr was observed except in the sample subjected to LED illumination in the presence of RF. Nevertheless, regarding the aggregative state observed in the SDS-PAGE results, it was not possible to discern if there were differences between the untreated S_CTRL compared to S_LED and S_LED_RF that were subjected to photo-oxidation. Based on the molar amounts, the amount of di-Tyr formed, even under the most drastic conditions (in the presence of RF), was much lower than that of the LAL already present in the NaCas before irradiation (0.03:1 compared to 0.1:1). The absence of an increase in aggregates suggests that the di-Tyr crosslinks formed between casein subunits that had already aggregated together through LAL crosslinks, probably through exploiting the proximity of the amino acids involved.

### 3.4. Film Characterization

#### 3.4.1. Thickness of Films from Functionalized NaCas

The thickness of the functionalized NaCas films did not vary across the different treatments (*p* > 0.05) (Table 3). In a previous study, we prepared NaCas samples with different fat contents and observed that this parameter influenced the thickness of the derived films, among other characteristics [7]. Therefore, the fat content of the powder was kept constant in the present study to determine the specific effects of casein functionalization on inducing crosslink formation.

#### 3.4.2. FTIR Analysis of Functionalized NaCas Films

**Figure 3:**
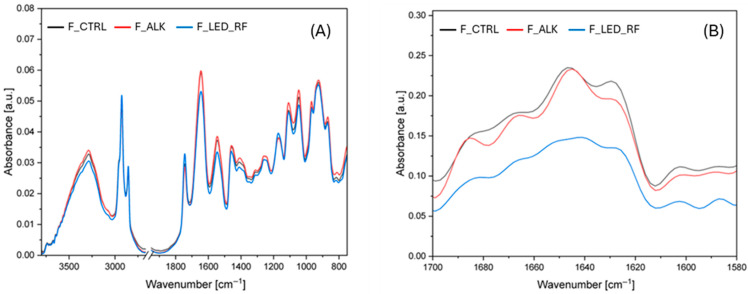
FTIR spectra of the NaCas films: (**A**) Absorbance spectra of the films in the regions 3800–2750 cm^−1^ and 1900–750 cm^−1^. (**B**) Fourier-deconvoluted spectra of Amide I region (1700–1600 cm^−1^).

FTIR spectroscopy was performed to investigate the chemical changes in the NaCas films induced by the functionalization. The spectra of the different films are shown in Figure 3A with focus on the most relevant wavenumber ranges. For clarity of the spectra, it was decided not to show the spectra of F_LED.

Caseins typically show absorption bands at 3288 cm^−1^ due to stretching vibrations of O-H and N-H, while the peak at 2926 cm^−1^ is related to the stretching vibrations of -CH_2_ groups [16,37] (Figure 3A). In this spectral region, no significant shifts in the O–H or –CH_2_ stretching bands were detected. The peak at 1644 cm^−1^ is attributed to the Amide I band, mainly due to C=O and C-N stretching vibrations. Peaks in the 1550–1510 cm^−1^ range correspond to the Amide II band, which is associated with N–H bending and C–N stretching vibrations [16,38,39]. Finally, the bands around 1455 cm^−1^ can be attributed to the symmetric stretching vibrations of the carboxylate group (COO^−^) [37], while the bands between 1240 and 1260 cm^−1^ were assigned to the Amide III region, which involves C–N stretching and N–H bending vibrations. In the NaCas films, a characteristic band was observed at 1110 cm^−1^, while additional bands in the 925 to 970 cm^−1^ range were attributed to interactions involving monocationic Na^+^ [40]. Furthermore, typical bands associated with the presence of glycerol in films (between 800 and 1150 cm^−1^) were also present in this region [6], reflecting strong binding interactions between NaCas and the hydroxyl groups of glycerol.

The Amide I region of a protein’s infrared spectrum is considered the most sensitive indicator of changes in secondary structure. Figure 3B shows the Fourier-deconvoluted spectra of the samples, while Table 4 summarizes the effects of the alkaline and LED treatments, expressed as the percentage contribution of the Amide I region components.

The results showed that the alkaline treatment led to a decrease in the β-turn and β-sheet contents, accompanied by a significant increase in the proportion of random coils. As suggested by Zhang et al. [41], alkaline treatment of proteins promotes a reduction in β-regions and an increase in random coils. These findings are also consistent with those of Markoska et al. [27], who observed a slight reduction in β-turn and β-sheet contents in β-casein at pH 7 and 20 °C. Moreover, the alkaline treatment appeared to promote the formation of intermolecularly aggregated β-sheets, likely due to enhanced crosslinking. This is clearly reflected in Table 5, where the most significant peak shift towards higher wavenumbers was observed between 1682 and 1699 cm^−1^, particularly in the F_ALK sample. Interestingly, β-sheet aggregates increased with rising levels of LAL, which further supports the hypothesis of crosslink-driven aggregation. The role of LAL in the formation of intermolecular crosslinks is highlighted by the electrophoretic trace of the F_ALK sample (Figure 2), which showed a notable reduction in the monomeric forms and the accumulation of high-molecular-weight aggregates. Therefore, it can be hypothesized that the decrease in intramolecular β-sheet and β-turn structures, along with the increase in random coils and intermolecular β-sheet aggregates, results from conformational changes induced by the alkaline treatment through the formation of LAL. Under alkaline conditions, the native secondary structures of casein appear to be destabilized due to the high pH and chemical modifications to its side chains, which likely disrupt intramolecular hydrogen bonding. This leads to partial unfolding of the protein and an increase in disordered regions, as indicated by the enhanced presence of random coils. At the same time, the exposure of previously buried reactive groups promotes the formation of intermolecular crosslinks, resulting in the assembly of extended β-sheet aggregates. These aggregates are structurally distinct from native β-sheets and suggest enhanced protein–protein interactions, possibly stabilized by covalent bonds such as those mediated by LAL. As a consequence, the overall structural profile of casein shifts from a compact, intramolecular organization to a more extended, aggregated arrangement, consistent with the spectroscopic and electrophoretic data.

In contrast to the structural changes observed following the alkaline treatment, the modifications induced by light exposure in the F_LED_RF sample showed a different pattern. Specifically, a decrease in the β-sheet content and an increase in random coil structures were observed (Table 4), without a corresponding rise in β-sheet aggregates, which was also confirmed by the electrophoretic profile shown in Figure 2. The increase in random coil structures was further supported by the significant shift in the band at 1645 cm^−1^ towards lower wavenumbers in the F_CTRL sample (1640 cm^−1^ in the F_LED_RF sample). This behavior was observed by Haddad-Khoozani and Soltanizadeh [42] after energetic treatment of a NaCas solution with plasma in the presence of polysaccharides, which led to the formation of conjugates.

In the F_LED_RF sample, the shift in the tyrosine-related band from 1171 to 1174 cm^−1^, together with the shift in the tryptophan-associated band from 1459 to 1461 cm^−1^, suggests overall conformational changes in the protein structure induced by LED light exposure [43]. Notably, since di-Tyr formation is known to alter the vibrational environment of the C–OH group in tyrosine—particularly in the 1170 cm^−1^ region—this shift may be associated with increased di-Tyr crosslinking (Table 2) [44]. Therefore, light exposure in the presence of RF can effectively enhance crosslinking along the protein chains, introducing both rigid β-sheet domains and flexible di-Tyr networks.

#### 3.4.3. Mechanical Properties of the Films

**Figure 4:**
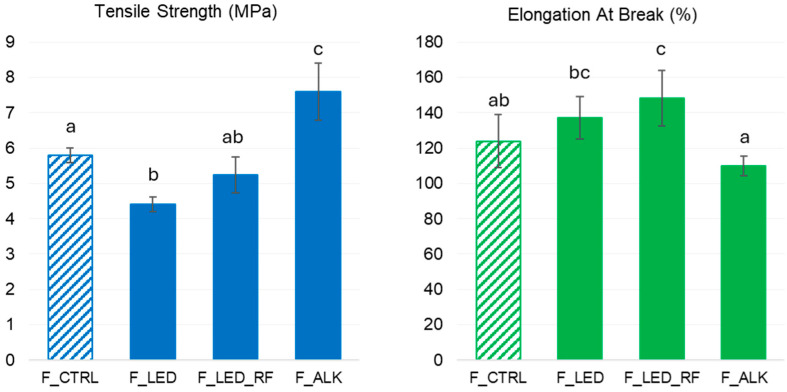
Tensile strength (TS) and elongation at break (EAB) of the experimental films. F_CTRL: control film (non-functionalized); F_LED: film obtained from NaCas solution exposed to LED light; F_LED_RF: film obtained from NaCas solution exposed to LED light in presence of riboflavin; F_ALK: film obtained from NaCas solution heat-treated at alkaline pH. Results are expressed as mean ± standard deviation of 3 replicates. Samples with different superscript letters are significantly different (*p* < 0.05) according to Tukey’s test.

The changes in tensile strength (TS) and elongation at break (EAB) were measured to evaluate the effects of the functionalization treatments on the mechanical properties of the films. TS refers to the maximum stress a film can withstand before failure and provides insights into key attributes such as fracture resistance and structural integrity. EAB, expressed as the percentage increase in the film’s original length, reflects its ductility and indicates the extent of deformation the film can undergo before breaking. Depending on the intended application, a stiffer or more deformable film may contribute to better packaging performance. The measured values are shown in Figure 4.

In a recent study, we observed that both parameters in neat NaCas films were significantly affected by the amount of milk fat retained in the NaCas and by the degree of fat dispersion (due to the sonication method for the film-forming solution: in a bath or using an immersion probe) [7]. Therefore, both the fat content and sonication mode were the same for all treatments in the present study to determine how the functionalization treatments alone influenced the mechanical properties of the films.

The F_ALK film, obtained from the NaCas with the highest concentration of LAL (3540 µg/g protein) and with no di-Tyr, showed the highest TS values and the lowest EAB values. These results are in accordance with the presence of large covalent aggregates observed in the corresponding SDS-PAGE (Figure 2) and FTIR (Figure 3) results, suggesting that LAL crosslinking induced a denser protein network in NaCas heated at pH 9, which ultimately enhanced film stiffness. In contrast, the F_LED and F_LED_RF films obtained from NaCas exposed to light showed lower TS values and higher EAB values compared to the F_ALK film. Unexpectedly, the simple exposure of the NaCas solution to light caused a decrease in TS and an increase in EAB in the F_LED film despite almost no di-Tyr forming, suggesting the role of other light-induced modifications to casein. The formation of di-Tyr (501 µg/g protein) in S_LED_RF due to the addition of RF resulted in small but significant increases in the TS and EAB values in F_LED_RF compared to F_LED. Such a limited impact of di-Tyr on the mechanical properties is likely due to its lower content (in terms of molar ratio with casein) compared to LAL (0.03:1 compared to 0.1:1). The SDS-PAGE results showed that the formation of di-Tyr occurred in NaCas that had already been partly crosslinked due to the presence of a certain amount of LAL. Furthermore, the effects on the mechanical properties of protein photo-oxidation vs. protein crosslinking will be investigated in the future. To the authors’ knowledge, no previous studies have explored this aspect in NaCas solutions.

## 4. Conclusions

The present study investigated the effectiveness of two green functionalization approaches in improving selected properties of NaCas films. Alkaline thermal treatment promoted LAL formation and produced covalent aggregates that were able to strengthen the protein network. The derived film had an increased TS and reduced EAB. In contrast, exposure of NaCas to LED light after the addition of RF significantly increased di-Tyr production compared to the control samples. This functionalization enhanced the film’s elasticity and plasticity, as evidenced by the increased EAB values. The combined effects of these two different protein crosslinking approaches demonstrated that this could be an innovative strategy for broadening the functionality of NaCas films tailored to specific green and sustainable applications. It should also be mentioned that other covalent crosslinks, such as isopeptide bonds, might have also formed and contributed to the observed changes.

Moreover, the proposed NaCas-based material, synthesized without any additives and with very low levels of crosslinks, could be used in films in contact with foods or as a basis for producing edible films since the crosslinking compounds are firmly bound within the protein matrix and are not likely to migrate or be released. The biodegradability of such functionalized films is worth investigating to support their industrial application.

## Figures and Tables

**Table 1:** Levels of LAL in the experimental NaCas samples.

Sample	LAL (µg/g Protein)	LAL (µmol/g Protein)
S_CTRL	867 ± 22 ^a^	3.88
S_LED	907 ± 27 ^b^	4.06
S_LED_RF	722 ± 11 ^c^	3.46
S_ALK	3540 ± 64 ^d^	15.86

Results are expressed as mean ± standard deviation of 3 replicates. Data in the same column followed by different superscript letters are significantly different (*p* < 0.05) according to the Tukey test.

**Table 2:** Levels of di-Tyr in the experimental NaCas samples.

Sample	di-Tyr (µg/g Protein)	di-Tyr (µmol/g Protein)
S_CTRL	n.d.	n.d.
S_LED	4 ± 0.1	0.0111
S_LED_RF	501 ± 7.5	1.39
S_ALK	1 ± 0.0	0.0003

Results are expressed as mean ± standard deviation of 3 replicates. n.d.: not determined.

**Table 3:** Thicknesses of experimental films.

Sample	Thickness (µm)
F_CRTL	145 ± 30 ^a^
F_LED	145 ± 14 ^a^
F_LED_RF	152 ± 15 ^a^
F_ALK	142 ± 4 ^a^

Thickness values are expressed as mean ± standard deviation of 3 replicates. Data in the same column followed by different superscript letters are significantly different (*p* < 0.05) according to the Tukey test.

**Table 4:** Effects of different treatments on the side chain and secondary structure of NaCas in the derived films.

Band Position (cm^−1^)	Band Assignment	F_CTRL (%)	F_ALK (%)	F_LED_RF (%)
1600–1610	Side chain	7.1 ± 0.6 ^a^	7.0 ± 0.7 ^a^	6.9 ± 0.2 ^a^
1615–1635	β-sheet	23.6 ± 2.5 ^a^	17.1 ± 2.7 ^b^	19.7 ± 1.5 ^bc^
1640–1645	Random coil	6.0 ± 1.0 ^a^	10.1 ± 1.6 ^b^	9.3 ± 1.3 ^b^
1645–1660	α-Helix	22.8 ± 2.5 ^a^	23.9 ± 3.2 ^a^	23.4 ± 2.9 ^a^
1660–1682	β-turn	25.3 ± 1.5 ^a^	21.8 ± 1.7 ^b^	25.5 ± 1.9 ^a^
1682–1699	Aggregated β-sheet	15.2 ± 3.1 ^a^	20.0 ± 4.7 ^b^	15.2 ± 2.8 ^a^

Data are presented as the average percentage area of each deconvoluted band ± standard deviation of 3 replicates. Data in the same column followed by different superscript letters are significantly different (*p* < 0.05) according to the Tukey test.

**Table 5:** Shifts in wavenumber in the FTIR spectra of the film samples.

Band Position (cm^−1^)	Band Assignment	F_CTRL	F_ALK	F_LED_RF
1615–1635	β-sheet	1629	1625	1628
1640–1645	Random coil	1647	1645	1640
1660–1682	β-turn	1667	1666	1667
1682–1699	Aggregated β-sheet	1683	1686	1682
1470–1450	Tryptophan	1459	1459	1461
1190–1155	Tyr/di-Tyr	1171	1171	1174

## Data Availability

The original contributions presented in the study are included in the article and Supplementary Materials. Further inquiries can be directed to the corresponding author.

## Supplementary Materials

The following supporting information can be downloaded at https://www.mdpi.com/article/10.3390/foods14162764/s1. Figure S1: Equipment for exposing NaCas solutions to LED light (A), and spectral irradiance of the LED light lamp used in the experiment (B); Figure S2: HPLC chromatograms used for di-Tyr determination. S_CTRL: control solution (non-functionalized); S_LED: NaCas solution exposed to LED light; S_LED_RF: NaCas solution exposed to LED light in presence of riboflavin; S_ALK: NaCas solution heat-treated under alkaline conditions; Table S1: Gross composition (g/100 g) of the prepared NaCas powder; Table S2: Densitometric analysis of the monomeric casein bands in Figure 2.

## Abbreviations

The following abbreviations are used in this manuscript:

FTIR	Fourier-transform infrared spectroscopy
TS	Tensile strength
EAB	Elongation at break
