# Supplementary material for: Functionalization of Sodium Caseinate for Production of Neat Films: Effects of Casein Crosslinking Induced by Heating at Alkaline pH or Light Exposure
Source: Foods. 2025 Aug 8;14(16):2764. doi: 10.3390/foods14162764 (PMC12386004; doi:10.3390/foods14162764)
Supplement: Supplementary file 1 [file foods-14-02764-s001.zip › foods-3754991-supplementary.pdf]

Supplementary Materials

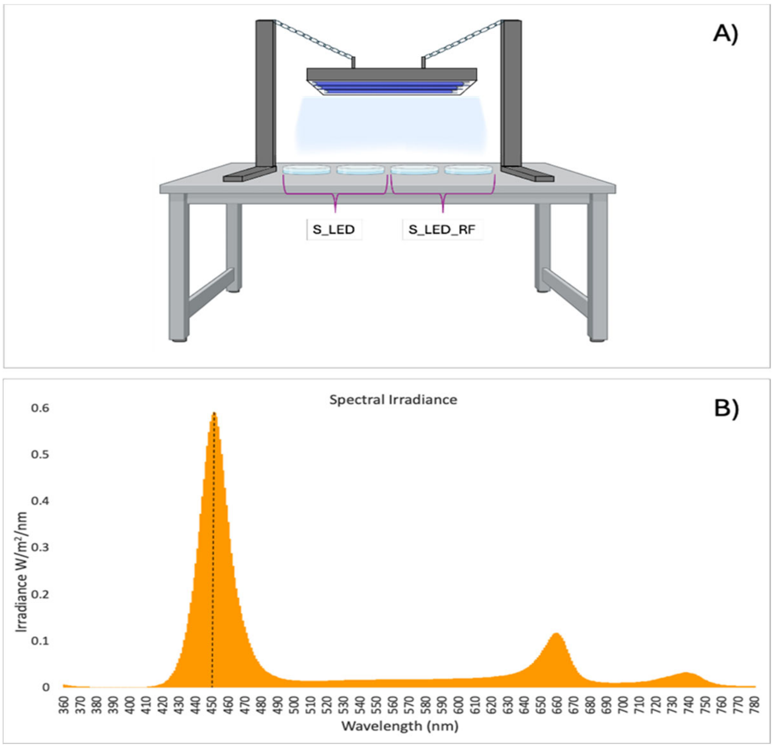

Figure S1. Equipment for exposing NaCas solutions to LED light (**A**), and spectral irradiance of the LED light lamp used in the experiment (**B**).

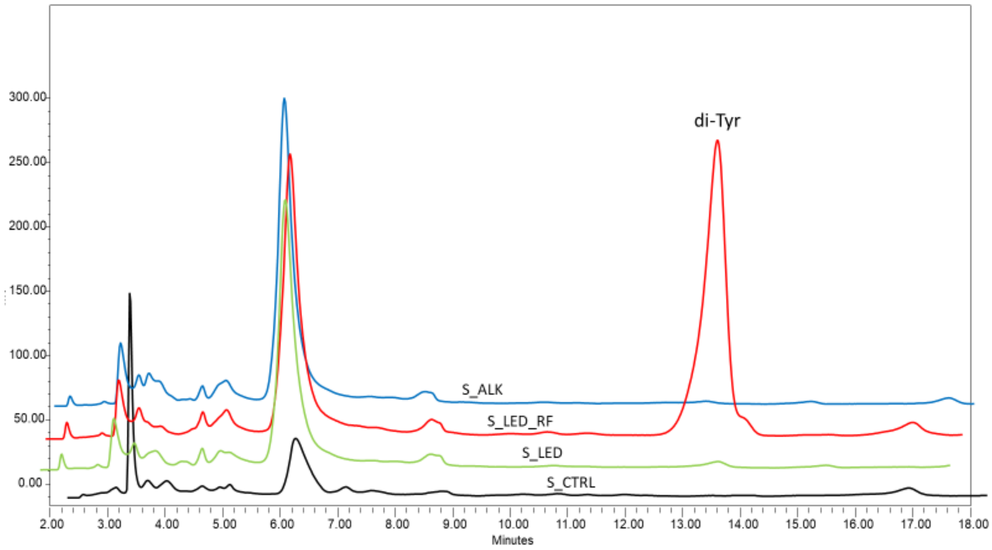

Figure S2. HPLC chromatograms used for di-Tyr determination. S_CTRL: control solution (non-functionalized); S_LED: NaCas solution exposed to LED light; S_LED_RF: NaCas solution exposed to LED light in presence of riboflavin; S_ALK: NaCas solution heat-treated under alkaline conditions.

Table S1. Gross composition (g/100 g) of the prepared NaCas powder.

Protein	Fat	Moisture	Ash
69.34 ± 0.70	23.42 ± 0.03	3.65 ± 0.10	3.46 ± 0.09

Table S2. Densitometric analysis of the monomeric casein bands in Figure 2.

Sample	Band volume (arbitrary units)	Relative band volume (%)
Casein	2990	100.0
S_CTRL	2254	75.4
S_LED	2022	67.6
S_LED_RF	2076	69.4
S_ALK	1377	46.1
